# High‐throughput Photoactive Magnetic Microrobots for Food Quality Control

**DOI:** 10.1002/smtd.202401952

**Published:** 2025-03-11

**Authors:** Roberto Maria‐Hormigos, Carmen C. Mayorga‐Martinez, Jeonghyo Kim, Martin Pumera

**Affiliations:** ^1^ Future Energy and Innovation Laboratory Central European Institute of Technology Brno University of Technology (CEITEC‐BUT) Purkyňova 123 Brno 61200 Czech Republic; ^2^ Advanced Nanorobots and Multiscale Robotics Lab Faculty of Electrical Engineering and Computer Science VSB–Technical University of Ostrava 17. listopadu 2172/15 Ostrava 70800 Czech Republic; ^3^ Department of Chemical and Biomolecular Engineering Yonsei University 50 Yonsei‐ro Seodaemun‐gu Seoul 03722 South Korea; ^4^ Department of Medical Research China Medical University Hospital China Medical University No. 91 Hsueh‐Shih Road Taichung 40402 Taiwan

**Keywords:** food analysis, microrobots, nanorobots

## Abstract

Ensuring food quality and safety according to stringent global standards requires analytical procedures that are accurate, cost‐effective, and efficient. This present innovative high‐throughput microrobots designed for the detection of antioxidants in food samples. These microrobots consist of photocatalytic bismuth subcarbonate anchored on silica‐coated magnetite nanoparticles. Upon exposure to UV light, they generate reactive oxygen species via photocatalysis, which oxidize the colorless dye into a green‐colored radical cation. The presence of antioxidants inhibits this reaction, allowing for the quantification of antioxidant activity. The magnetic Fe₃O₄/SiO₂ core enables steering of the microrobots using a transverse rotating magnetic field, facilitating automated assays on a custom‐designed 3D‐printed sensing platform. This results demonstrate that these magneto‐photocatalytic microrobots can perform automated, high‐throughput assessments of food quality, representing a significant advancement in food analysis technology.

## Graphical abstract

Magneto‐photocatalytic Bi₂O₂CO₃@Fe₃O₄ microrobots enable automated antioxidant detection in food. Under UV light, they generate reactive oxygen species that oxidize a colorless dye into a green radical cation, with antioxidants inhibiting dye oxidation. A rotating magnetic field steers these microrobots across a custom 3D‐printed platform, providing high‐throughput, cost‐effective, and accurate food quality assessments—offering a significant advance in automated food safety technology.

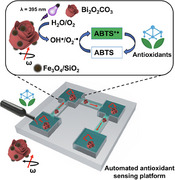

## 1 Introduction

Food quality frauds have risen in recent decades as a result of the globalization of the food industry. Fast and reliable procedures for food analysis are required to fulfill the increasing worldwide demand for high quality, safety controls, and no fraud due to emerging economies.^[^ ^1^ ^]^ Moreover, automation is very convenient to (1) minimize human errors during the analysis, (2) reduce cost, (3) increase productivity, and (4) optimize a part of a bigger process.^[^ ^2^ ^]^ Micro/nanorobots are micro/nanoscale materials that can convert an energy input into self‐motion in solution.^[^ ^3^, ^4^, ^5^, ^6^ ^]^ This motion enhances catalytic, photocatalytic, and biorecognition reactions “on‐the‐fly” by mass transfer enhancement.^[^ ^5^, ^6^ ^]^ For these reasons, several bio‐sensing applications have been developed using micro/nanorobots in recent years.^[^ ^7^, ^8^, ^9^, ^10^ ^]^ Moreover, they are highly compatible with automation, especially magnetically driven micro/nanorobots.^[^ ^11^, ^12^, ^13^ ^]^ In addition, magnetically driven micro/nanorobots do not produce substrate alterations during their motion, which makes them ideal for food applications.^[^ ^14^, ^15^, ^16^ ^]^ Even though biosensing has been achieved in several magnetic microrobots studies, their performances are still quite limited in comparison over current methodologies. To date, nanozyme‐based microrobots have been explored for food quality control applications in only a few instances. Rojas et al. developed a magnesium/gold Janus catalytic microrobot capable of catalyzing the hydrolysis of diphenyl phthalate in food, facilitating its subsequent electrochemical detection.^[^ ^17^ ^]^ Similarly, Luo et al. reported a Janus catalytic microrobot designed to enhance arsenic extraction from rice using H_2_O_2_ as fuel.^[^ ^18^ ^]^ However, these approaches primarily served as tools for “sample preparation” in food quality control applications without directly participating in the detection assay. In this study, we introduce microrobots that not only assist in food quality assays but also actively perform the chemical reactions necessary for detection. On the other hand, previous food quality control applications utilizing microrobots primarily relied on “on‐the‐fly” biosensing strategies that depend on expensive biorecognition elements and complex fluorescence‐based detection methods. These approaches are less practical compared to fast and cost‐effective colorimetric assays, which are better suited for food quality control.^[^ ^14^, ^19^, ^20^ ^]^ This study shows a comprehensive comparative analysis on different samples with existing methods for antioxidant determination, which is crucial for justifying the adoption of a new technology. Antioxidants determination is highly relevant in food quality and fraud analysis due to the positive effects of antioxidants on human health, especially in the beverage industry.^[^ ^21^, ^22^ ^]^ Normally, a higher antioxidant concentration in the food substrate is related to higher‐quality products.^[^ ^23^ ^]^

**Scheme 1:**
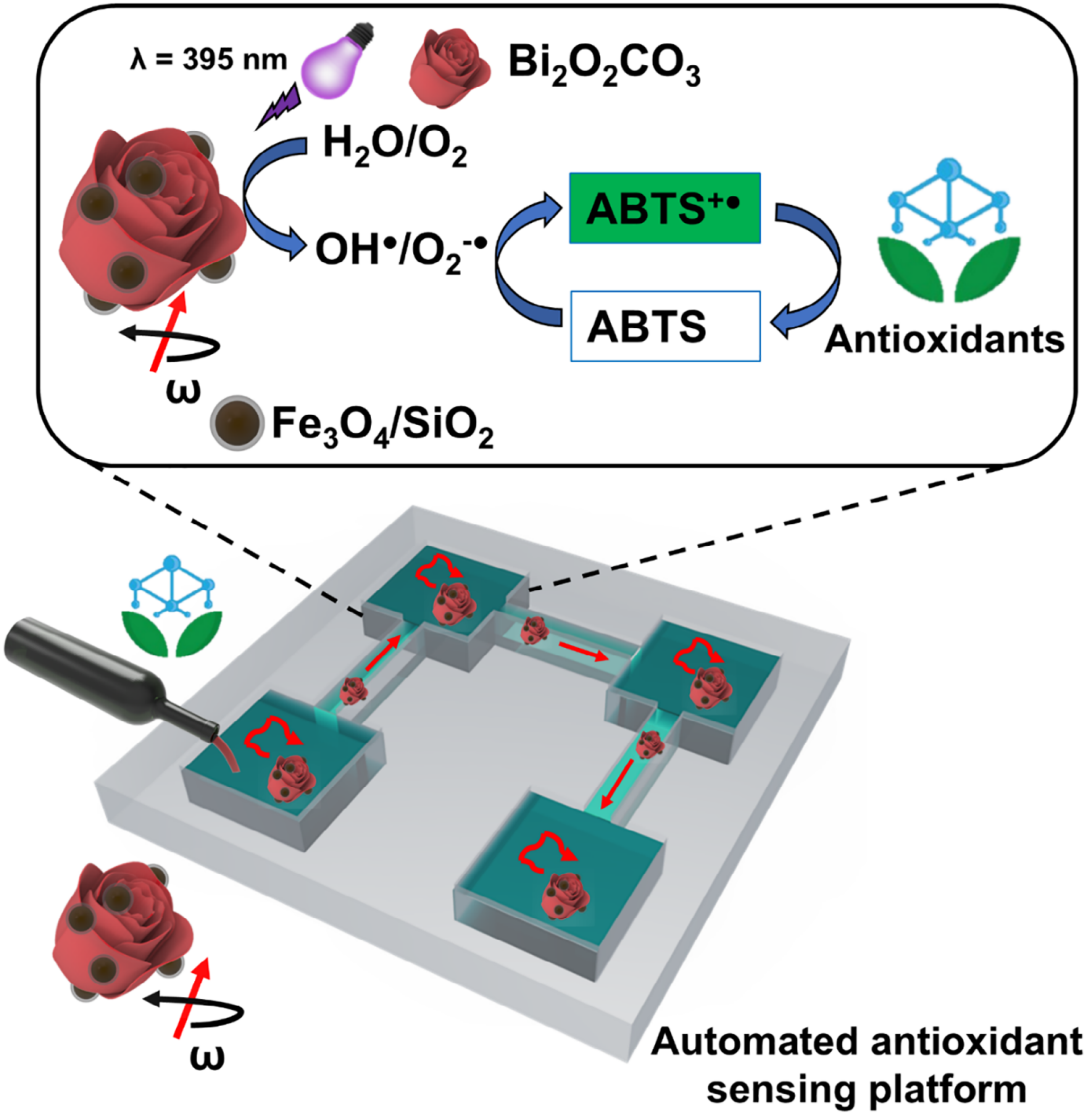
Schematic representation of antioxidant determination by a colorimetric assay based on photocatalytic magnetic microrobots in a fluidic platform for automation. Beverage samples rich in antioxidants are added to the reservoirs with the ABTS probe. Inset: ABTS colorimetric reaction mechanism employed for the antioxidant activity assay. Photocatalytic magnetic Bi_2_O_2_CO_3_@Fe_3_O_4_ microrobots navigate inside the sensing platform via an automated magnetic field to perform the photocatalytic reaction in each reservoir.

In this work, a magnetic Bi_2_O_2_CO_3_@Fe_3_O_4_ microrobot with enhanced photocatalytic activity was developed to carry out high‐throughput food quality control. Bi_2_O_2_CO_3_ microparticles were decorated with silica‐coated Fe_3_O_4_ nanoparticles, where the silica shell provides a strong negative surface charge due to the abundance of hydroxyl (─OH) groups, facilitating successful electrostatic assembly between the two materials and enabling magnetically propelled motion under a rotating magnetic field. The magnetic microrobots’ motion amplifies the diffusion necessary for effective mixing in the antioxidant activity assay and the automation of the analytical procedure within the homemade 3D‐printed platform (**Scheme** **1**). As can be seen, Bi_2_O_2_CO_3_@Fe_3_O_4_ microrobots performed the antioxidant activity assay in each reservoir of the 3D‐printed platform and were guided between the reservoirs using a rotating magnetic field. This magnetic field was programmed to minimize human interaction in the automated assay. Antioxidant assay is based on the oxidative radicals (OH^·^/O_2_^−·^) photogenerated by Bi_2_O_2_CO_3_ under UV light irradiation and used to oxidize colorless 2,2'‐azino‐bis(3‐ethylbenzothiazoline‐6‐sulfonic acid) (ABTS) into green‐colored ABTS^+·^.^[^ ^24^, ^25^, ^26^ ^]^ On the presence of antioxidants in the food substrate, this green‐colored formation is hampered following the mechanism shown in Scheme 1.

## 2 Results and Discussion

### 2.1 Synthesis and Characterization of Magneto‐Photocatalytic Bi_2_O_2_CO_3_@Fe_3_O_4_ Microrobots

The magneto‐photocatalytic Bi_2_O_2_CO_3_@Fe_3_O_4_ microrobots were fabricated using an electrostatic assembly in which negatively stabilized silica‐coated Fe_3_O_4_ nanoparticles were adhered to the surface of positively charged Bi_2_O_2_CO_3_ microparticles. Bi_2_O_2_CO_3_ is a well‐known photocatalyst that can produce radicals such as OH^·^ and O_2_^−·^ under UV light irradiation due to their band gap of between ≈+2.7 V and −0.7 V versus normal hydrogen electrode (NHE) (OH^−^/OH^·^, +1.99 V and O_2_/O_2_^−·^ −0.13 V vs NHE).^[^ ^27^ ^]^ These radicals were employed in this work to develop an antioxidant activity assay based on ABTS oxidation by these radicals and the radicals’ scavengers contained in the samples in order to hamper such reaction. The photocatalytic Bi_2_O_2_CO_3_ microparticles were synthesized by a previously reported hydrothermal method (details in the Experimental Section).^[^ ^28^ ^]^ The Fe_3_O_4_/SiO_2_ nanoparticles, serving as the magnetic component in microrobots, were prepared by subsequent solvothermal and sol‐gel processes following the previously reported procedures (details in the Experimental Section).^[^ ^29^, ^30^, ^31^ ^]^ Encapsulation by a thin silica (SiO_2_) layer surrounding the nanoparticles provides a strong negative surface charge due to the presence of abundant hydroxyl (─OH) groups. Finally, through a straightforward overnight mixing and stirring process, the Fe_3_O_4_/SiO_2_ magnetic particles were electrostatically attached to the surface of Bi_2_O_2_CO_3_ microparticles, resulting in the formation of hybridized Bi_2_O_2_CO_3_@Fe_3_O_4_ microrobots.

Moreover, the morphology and elemental composition of bare Fe_3_O_4_ and Fe_3_O_4_/SiO_2_ nanoparticles were characterized by transmission electron microscopy (TEM) and energy‐dispersive spectrometry (EDS) mapping. Bare Fe_3_O_4_ nanoparticles showed a spherical shape with sizes ≈250 nm and composed of small Fe_3_O_4_ nanocrystals (Figure S1A, Supporting Information).^[^ ^29^, ^30^ ^]^ After the coating process, a thin layer of SiO_2_ shell with a smooth surface was covered over the Fe_3_O_4_ nanoparticles (Figure S1B, Supporting Information). The EDS mapping images further corroborated the structural distribution of elemental Fe, O, and Si over the nanoparticles. As shown in Figure S1C (Supporting Information), the Si signal is concentrated in the nanoparticles’ edges, meanwhile, the Fe signal is concentrated in the core and the O signal is homogeneously distributed along the nanoparticles, confirming their core‐shell structure.

**Figure 1:**
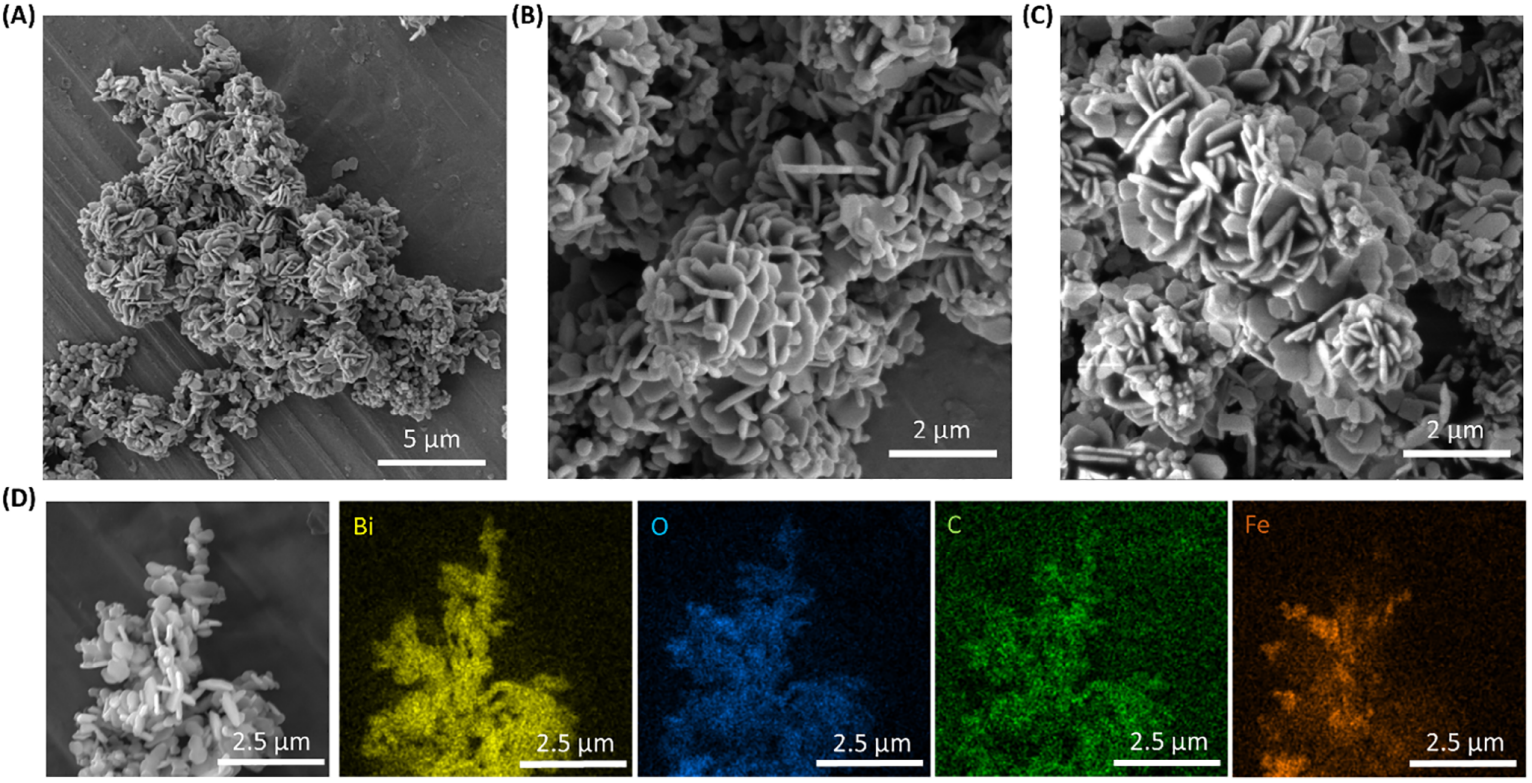
Structural characterization. A) SEM image of Bi_2_O_2_CO_3_@Fe_3_O_4_ microrobots. B) Flower‐like structures magnification. C) SEM image of Bi_2_O_2_CO_3_ microparticles without Fe_3_O_4_. D) EDS spectrum analysis of a microrobot surface.

Bi_2_O_2_CO_3_@Fe_3_O_4_ microrobots’ morphology and chemical composition were characterized by scanning electron microscopy (SEM) and EDS elementary mapping from SEM images, respectively. **Figure** **1**A shows Bi_2_O_2_CO_3_@Fe_3_O_4_ microrobot agglomerates of flower‐like structures with sizes ≈4 µm (Figure 1B). Figure 1C shows Bi_2_O_2_CO_3_ microparticles without Fe_3_O_4_/SiO_2_ nanoparticles. This flower‐like structure corresponds to Bi_2_O_2_CO_3_ microparticles as previously reported and did not suffer any modification.^[^ ^27^ ^]^ Hence, Fe_3_O_4_ nanoparticles incorporation was confirmed by the presence of Fe in the microrobots’ EDS mapping (Figure 1D). The iron signal is concentrated in some areas, indicating the formation of some clusters in specific sites of the BiO_2_CO_3_ microparticles. Moreover, the EDS spectrum from Figure S2 (Supporting Information) shows the elemental composition of the microrobots which are composed of Bi, C, and O from Bi_2_O_2_CO_3_ and Fe and O from Fe_3_O_4_ nanoparticles as can be seen by their respective elemental signals in the mapping. Cu and Au elemental signals are observed owing to the copper tape substrate and gold sputter used for sample preparation.

**Figure 2:**
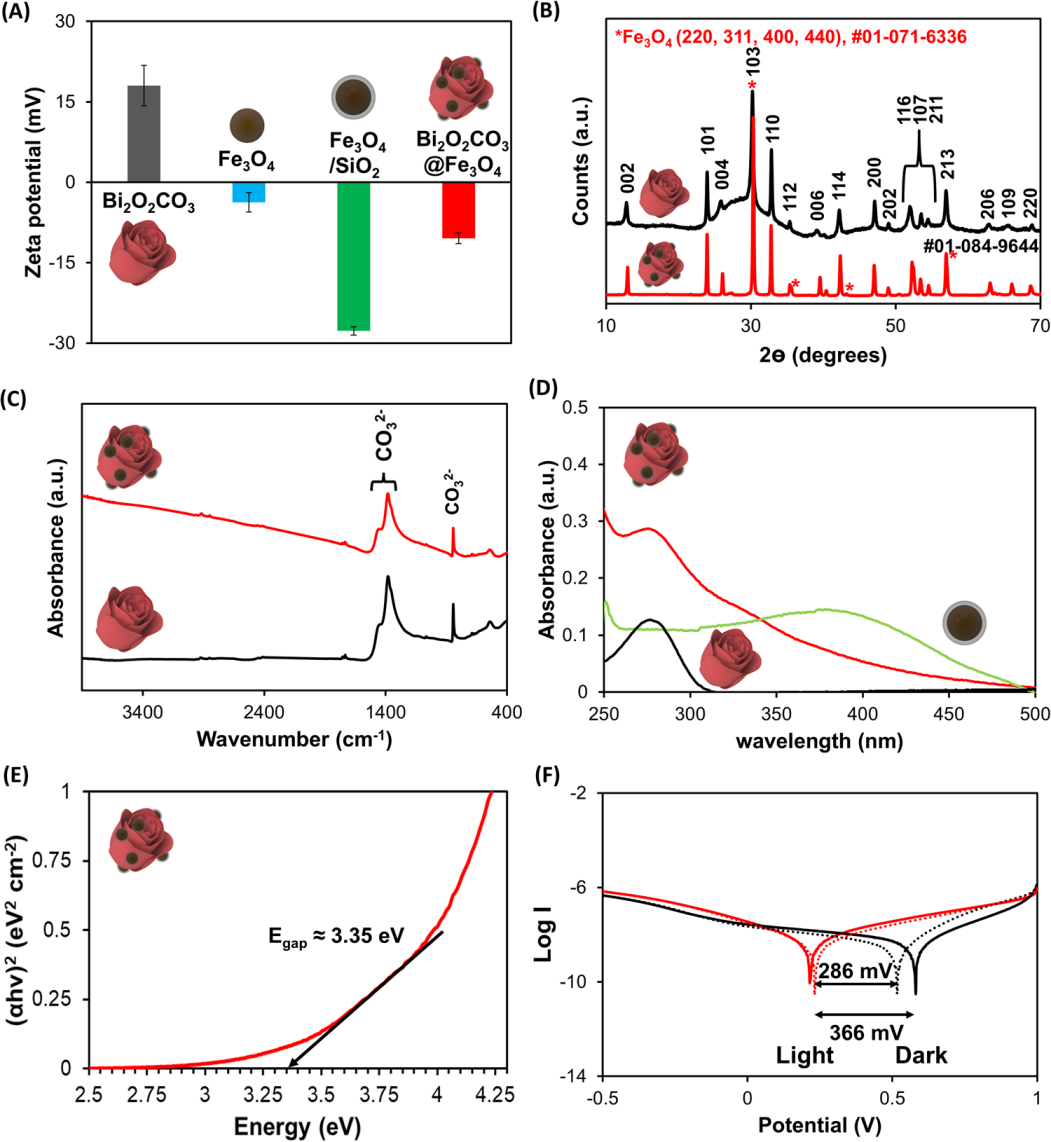
Magnetic photocatalytic Bi_2_O_2_CO_3_@Fe_3_O_4_ microrobots characterization. A) Zeta potential of Bi_2_O_2_CO_3_ microparticles, bare Fe_3_O_4_, Fe_3_O_4_/SiO_2_ nanoparticles, and Bi_2_O_2_CO_3_@Fe_3_O_4_ microrobots. B) XRD of Bi_2_O_2_CO_3_ particles and the microrobots. (Bi_2_O_2_CO_3_ 01‐084‐9644 and Fe_3_O_4_ 01‐071‐6336). C) FTIR characterization of Bi_2_O_2_CO_3_ particles and the microrobots. D) UV–Vis spectra of Bi_2_O_2_CO_3_, Fe_3_O_4_/SiO_2_ particles, and Bi_2_O_2_CO_3_@Fe_3_O_4_ microrobots. E) TAUC plot from UV–Vis spectra in Figure 2D. F) Tafel plots of Bi_2_O_2_CO_3_@Fe_3_O_4_ microrobots (solid line) and bare Bi_2_O_2_CO_3_ microparticles (dotted line), λ = 395 nm in water.

An electrostatic assembly of the magneto‐photocatalytic microrobots was designed based on their surface charge. **Figure** **2**A shows the zeta potentials of the individual micro/nanoparticles and the hybridized microrobots. Bi_2_O_2_CO_3_ microparticles exhibited a positive zeta potential of +18 ± 3.8 mV, whereas Fe_3_O_4_ covered with a SiO_2_ shell showed a negative surface charge of −27.7 ± 0.7 mV. However, the surface charge of pristine Fe_3_O_4_ was −4 ± 1.8 mV and, after modification with SiO_2_, became negative thanks to its hydroxyl functional group. Finally, the hybridized Bi_2_O_2_CO_3_@Fe_3_O_4_ microrobots showed a net surface charge of −10 ± 1 mV, indicating effective electrostatic interactions between the Bi_2_O_2_CO_3_ microparticles and the magnetic components.

Crystallographic information of Bi_2_O_2_CO_3_ microparticles and Bi_2_O_2_CO_3_@Fe_3_O_4_ microrobots was obtained by X‐ray diffraction (XRD) analysis. Figure 2B shows the XRD peaks of Bi_2_O_2_CO_3_ bismutite single‐crystal structure, confirming the successful synthesis of the photocatalytic material (ref. code 01‐084‐9644).^[^ ^32^ ^]^ Moreover, the XRD pattern of Bi_2_O_2_CO_3_@Fe_3_O_4_ microrobots showed a mixture of crystalline forms of Bi_2_O_2_CO_3_ and magnetite (Fe_3_O_4_, ref. code 01‐071‐6336).^[^ ^33^ ^]^ In addition, the semi‐quantitative XRD analysis of the peaks revealed a proportion of 9:1 (Bi_2_O_2_CO_3_:Fe_3_O_4_) between both materials. Fourier‐transform infrared (FTIR) characterization of Bi_2_O_2_CO_3_ microparticles and Bi_2_O_2_CO_3_@Fe_3_O_4_ microrobots confirmed the predominant Bi_2_O_2_CO_3_ composition on the microrobots. Figure 2C shows the FTIR bands at 1450, 1374, and 840 cm^−1^ of CO_3_^2−^ vibrations.^[^ ^32^ ^]^ However, the Fe─O band ≈560 cm^−1^ was not visible due to the low concentration of Fe_3_O_4_ on the microrobots.

The photocatalytic behavior of Bi_2_O_2_CO_3_@Fe_3_O_4_ microrobots was characterized by UV–Vis spectrophotometry. Figure 2D shows the UV–Vis spectra of Bi_2_O_2_CO_3_@Fe_3_O_4_ microrobots and their parts, Bi_2_O_2_CO_3_ and Fe_3_O_4_ nanoparticles. Microrobots had a broad absorption below 400 nm with a peak at 280 nm owing to the broad absorption of Fe_3_O_4_ nanoparticles below 400 nm and the absorption peak of bare Bi_2_O_2_CO_3_. TAUC plot representation from the UV–Vis spectra showed a band gap of 3.35 eV for the microrobots (Figure 2E). This value is in accordance with previous reports.^[^ ^27^ ^]^ Moreover, Fe_3_O_4_ band gap of a 2.15 eV and bare Bi_2_O_2_CO_3_ microparticles band gap of 3.85 eV was calculated from their respective TAUC plots (Figure S3, Supporting Information). The photocatalytic process was investigated by the mixed potential of the microrobots. Figure 2F shows the mixed potential of Bi_2_O_2_CO_3_@Fe_3_O_4_ microrobots under dark and light conditions. The mixed potential was shifted toward more anodic potentials of 366 mV due to the microrobots’ photocatalytic activity.^[^ ^34^ ^]^ Such photocatalytic activity is owing to Bi_2_O_2_CO_3_ microparticles as can be seen by the 286 mV mixed potential of the microparticles. A slightly lower mixed potential shift was observed in the bare Bi_2_O_2_CO_3_ microparticles compared to the microrobots owing to the slightly higher band gap (3.85 eV) which makes the bare material more difficult to excite under 395 nm light.

The photocatalytic activity mechanism of Bi_2_O_2_CO_3_@Fe_3_O_4_ composite materials has been reported previously.^[^ ^35^, ^36^, ^37^, ^38^ ^]^ Under 395 nm light irradiation, Bi_2_O_2_CO_3_ predominantly generates reactive oxygen species (ROS), such as OH^·^ and O_2_^−·^, in solution, allowing the determination of radical scavenging compounds such as polyphenolic antioxidants.

(1) $$Bi_{2}O_{2}CO_{3}+\mathrm{UV}\mathrm{light}\left(395\;\;\mathrm{nm}\right)\to Bi_{2}O_{2}CO_{3}\left(e^{-}\right)+Bi_{2}O_{2}CO_{3}\left(h^{+}\right)$$

(2) $$Fe_{3}O_{4}+\mathrm{Visible}\mathrm{light}\to Fe_{3}O_{4}\left(e^{-}\right)+Fe_{3}O_{4}\left(h^{+}\right)$$

(3)

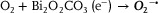

(4) $${O_{2}}^{-•}+H^{+}\to \mathrm{OO}H^{•}$$

(5)

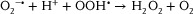



(6)

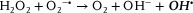




The formation of O_2_^−·^ and OH^·^ radicals by Bi_2_O_2_CO_3_ under light irradiation has been previously validated through electron spin resonance (ESR) and electron paramagnetic resonance (EPR), confirming the proposed mechanism.^[^ ^35^, ^36^, ^37^ ^]^

### 2.2 Magnetic Actuation of Bi_2_O_2_CO_3_@Fe_3_O_4_ Microrobots

**Figure 3:**
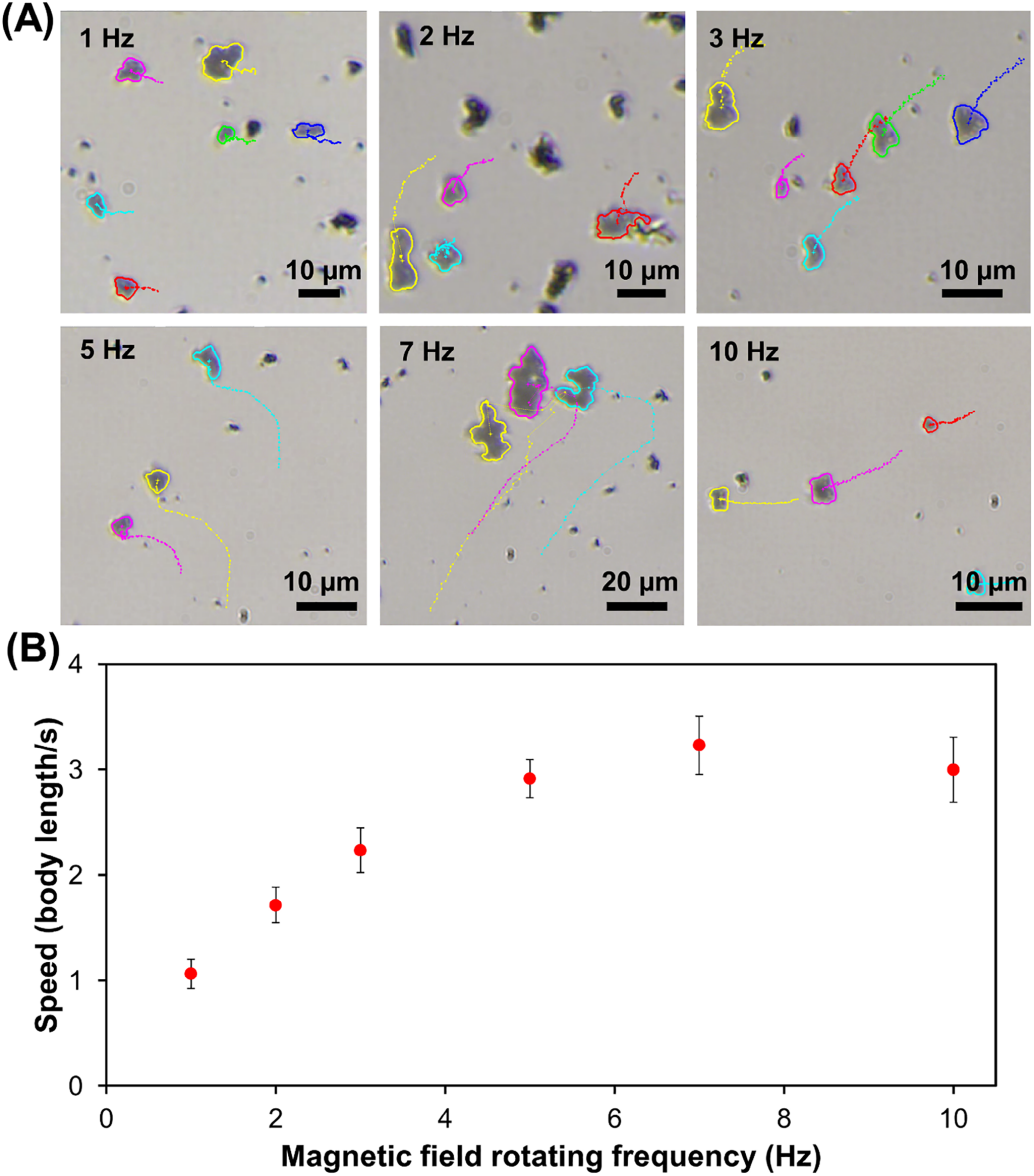
A) Time‐lapse images of Bi_2_O_2_CO_3_@Fe_3_O_4_ microrobots magnetic actuation and their trajectories under a rotating magnetic field at different rotating frequencies. B) Microrobots’ mean speed under a rotating magnetic field at different rotating frequencies (*n* = 10 microrobots). Magnetic field intensity: 5 mT.

Magnetic actuation followed a surface “walk” mechanism achieved via a transversal rotating magnetic field as previously reported by our group.^[^ ^39^ ^]^ **Figure** **3**A shows the trajectories of the microrobots for 2 s at different rotating frequencies of the magnetic field as captured in Videos S1–S6 (Supporting Information). As can be seen, there was a very wide size distribution among the microrobots, hence, their speed was corrected based on the length of the microrobots on the rotating axis (body length s^−1^). Figure 3B shows that the Bi_2_O_2_CO_3_@Fe_3_O_4_ microrobots’ speed increases with the rotating magnetic field frequency (*n* = 10 microrobots). However, at higher frequencies (7 and 10 Hz, see Videos S5 and S6, Supporting Information), rotation along the long axis combined with microrobots’ walking hampered their motion.^[^ ^40^ ^]^ The magnetic actuation of the microrobots was used to enhance and automate the antioxidation activity assay proposed in this work. Moreover, this magnetic “walking” motion mechanism is even effective on quite rough surfaces, which is interesting for their application on different scenarios such as in‐line pipes or cheap home‐made 3D platforms as in this study. The food industry requires rapid in situ or on‐demand analysis where magnetic actuation is easily implemented for microrobots’ actuation to carry out the food substrate analysis.

### 2.3 Antioxidant Activity Assay Based on Bi_2_O_2_CO_3_@Fe_3_O_4_ Microrobots

The antioxidant activity assay is based on the inhibition of the ABTS oxidation reaction in the presence of radicals’ scavengers. ABTS is one of the most common compounds used in antioxidant activity assays owing to its high solubility in water. So, ABTS is chosen for the colorimetric assays as they are going to be performed on beverage samples directly. ABTS oxidation into green‐colored ABTS^+·^ was carried out by photocatalytic microrobots under UV light irradiation. However, in the presence of radicals’ scavengers, such oxidation is hampered proportionally to their antioxidant activity, and, thereby, green color evolution in the solution is inhibited. The antioxidant activity assay, mainly ABTS oxidation using Bi_2_O_2_CO_3_@Fe_3_O_4_ microrobots under UV light irradiation, was optimized to ensure reproducible results. Figure S4A (Supporting Information) shows the ABTS^+·^ UV–Vis spectrum of patron solutions at different concentrations. As can be seen, the compound revealed three maximum absorption peaks at 660, 745, and 840 nm. The absorbance at 745 nm was employed for the antioxidant activity assay and this absorbance increased according to the Lambert–Beer law between 1 and 10 mM. Hence, 10 mM was employed in the antioxidant activity assay. Figure S4B (Supporting Information) shows microrobot concentration optimization by ABTS oxidation in static mode at a fixed time of 20 min, during which ABTS oxidation was not complete, to study the effect of photocatalytic material concentration on the oxidation rate. As can be seen, 0.25 mg mL^−1^ microrobot concentration produced the maximum ABTS oxidation rate with reproducible results. A higher concentration of the microrobots (0.5 mg mL^−1^) did not improve the ABTS oxidation rate. Figure S4C,D (Supporting Information) show ABTS oxidation over time under UV light irradiation using static microrobots and magnetic actuated microrobots (5 mT, 5 Hz), respectively. For total ABTS oxidation, 30 min of light irradiation using static/sedimented Bi_2_O_2_CO_3_@Fe_3_O_4_ microrobots was needed, whereas only 20 min were needed using magnetically actuated (dynamic) microrobots due to intermixing produced by the microrobots’ movement. Magnetic actuation of microrobots significantly boosts the intermixing of chemicals within solutions, thereby accelerating the degradation of harmful substances, optimizing extraction rates, and increasing the frequency of detection events.^[^ ^6^, ^41^, ^42^ ^]^ So, in our case, this results in a more rapid analysis than conventional sensors, leading to an interesting screening application for food safety control. Moreover, conventional sensors are static sensors limited by analyte chemical diffusion from the bulk to the sensor surface. However, the robots’ navigation enhances reaction rates through micromixing generated by their actuation, effectively preventing chemical diffusion of the antioxidants to the photocatalytic surface where radicals are generated or radicals’ diffusion to the bulk solution. In this context, the robots function as solution micro‐stirrers.

**Figure 4:**
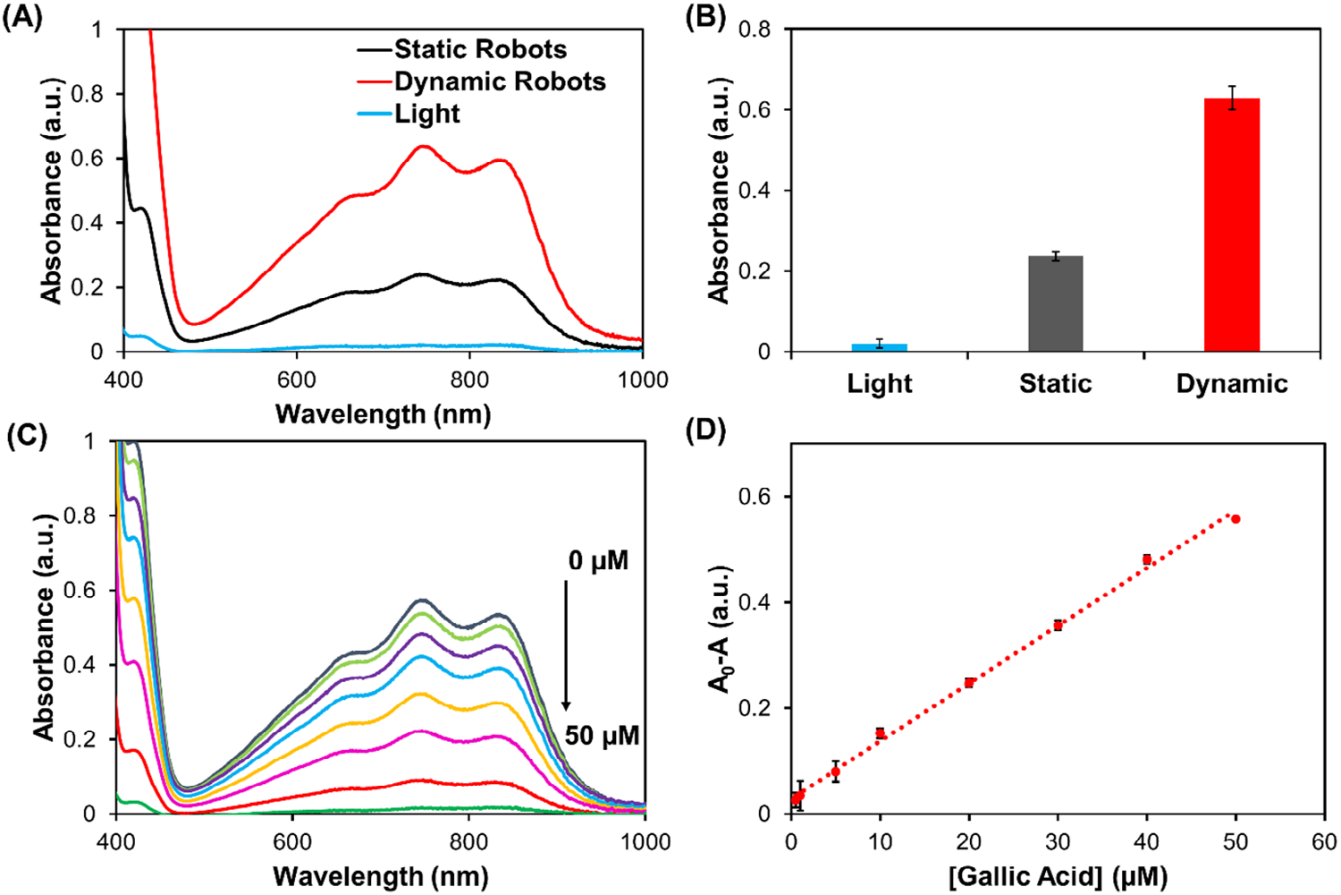
A) UV–Vis spectrum of ABTS (10 mM) oxidation in the absence (blue line) and presence of static (black line) as well as dynamic (red line) Bi_2_O_2_CO_3_@Fe_3_O_4_ microrobots after 20 min of light irradiation. B) Absorbance mean ± standard deviation at 745 nm from Figure 4A experimental conditions (*n* = 3). C) UV–Vis spectra of the antioxidant activity assay using gallic acid as a model compound. Experimental conditions: 10 mM ABTS after 20 min of light irradiation using 0.25 mg mL^−1^ of magnetically actuated Bi_2_O_2_CO_3_@Fe_3_O_4_ microrobots in the presence of 0, 1, 5, 10, 20, 30, 40, and 50 µM of gallic acid, Magnetic field: 5 mT, 5 Hz. D) Calibration plot of the antioxidant activity assay using gallic acid as model antioxidant compound (*n* = 3). Experimental conditions: same as Figure 4C, 10 mM ABTS, time = 20 min.

Under optimal conditions to perform the antioxidant activity assay (10 mM ABTS, 0.25 mg mL^−1^, 20 min UV light irradiation, under a rotating magnetic field 5 mT, 5 Hz), ABTS oxidation in the absence and presence of static and dynamic microrobots was compared (**Figure** **4**A). The UV–Vis spectra of the dynamic microrobots showed complete ABTS oxidation (A_750_ _nm_ = 0.63 ± 0.03, Figure 4B) compared to static microrobots (A_750_ _nm_ = 0.24 ± 0.01) while minimal ABTS oxidation was observed by only light without microrobots in the solution (A_750_ _nm_ = 0.02 ± 0.01). The ABTS^+·^ absorbance at 750 nm observed by the micromotors was equivalent to 10 mM ABTS^+·^ patron solution (Figure S4A, Supporting Information), indicating the reference value in the absence of radicals’ scavenger in the antioxidant activity assay (A_0_). Dynamic actuation of the microrobots accelerates ABTS^+·^ formation under light irradiation owing to the rapid diffusion of ABTS to the microrobots’ surface during their motion, which reduces the antioxidant activity assay overall time. An antioxidant activity assay was performed by ABTS oxidation reaction in the presence of the model antioxidant or sample solution (radicals’ scavengers) to measure the decrease in ABTS^+·^ absorbance with respect to the reference value (A_0_‐A). As beverage samples contain several antioxidant compounds, each with their own intrinsic antioxidant activities, a model antioxidant (gallic acid) was used to calibrate the antioxidant assay and to refer all sample results to gallic acid equivalent (GAE) antioxidant activity. Figure 4C shows the UV–Vis spectrum of antioxidant activity assay calibration using gallic acid. As can be seen, ABTS^+·^ absorbance decreased proportionally to gallic acid concentration between 1 and 50 µM while gallic acid above 50 µM inhibited totally ABTS oxidation and ABTS^+·^ absorption bands were no longer observed. Figure 4D shows the calibration plot of the antioxidant activity assay for gallic acid. The antioxidant activity assay based on Bi_2_O_2_CO_3_@Fe_3_O_4_ microrobots showed excellent analytical performance and linearity (*r* > 0.990) on the lineal range (2.8–50 µM) with a 0.9 µM limit of detection (LOD, 3x blank standard deviation criteria) and a 2.8 µM limit of quantification (LOQ, 10x blank standard deviation criteria). These low LOD and LOQ avoided sample intrinsic color interference during the colorimetric antioxidant activity assay by simple sample dilution. Moreover, the antioxidant activity assay based on Bi_2_O_2_CO_3_@Fe_3_O_4_ microrobots showed good sensitivity (0.011 µM^−1^) for quantitative analysis.

### 2.4 Automation and Sample Analysis

Antioxidant activity assay based on Bi_2_O_2_CO_3_@Fe_3_O_4_ microrobots was automated using a homemade 3D‐printed platform as a sensing platform. The sensing platform had four reservoirs connected by channels (width = 400 µm). The rotating magnetic field was programmed to make the microrobots follow a random trajectory in the reservoirs followed by lineal trajectories to transport the microrobots from one reservoir to another. This sequence was repeated three times and finished with the random motion in the last reservoir. Video S7 (Supporting Information) shows the Bi_2_O_2_CO_3_@Fe_3_O_4_ microrobots’ magnetic actuation inside of the sensing platform. As can be seen, the microrobots can move over the irregular surface of the 3D‐printed platform, overcoming the obstacles on the surface. Moreover, when microrobots followed a unidirectional trajectory, they followed the reservoir's walls until they went inside the channel that connects the reservoirs (Video S8, Supporting Information). This behavior allowed the transport of the microrobots between reservoirs without significant material losses. Finally, Video S9 (Supporting Information) shows the complete transportation of Bi_2_O_2_CO_3_@Fe_3_O_4_ microrobots from the first reservoir until the third by their magnetic actuation.

Analytical antioxidant determination assays were carried out in an automated way using an external controller for the magnetic field generator. The controller was first programmed to cause microrobots’ random motion in reservoir 1 for a fixed time. Then, a constant rotating magnetic field was applied to transport all the microrobots to reservoir 2 for another fixed time. This program was followed in the different reservoirs by changing the magnetic field by 90° to transport the magnetic microrobots between the reservoirs.^[^ ^39^ ^]^

**Figure 5:**
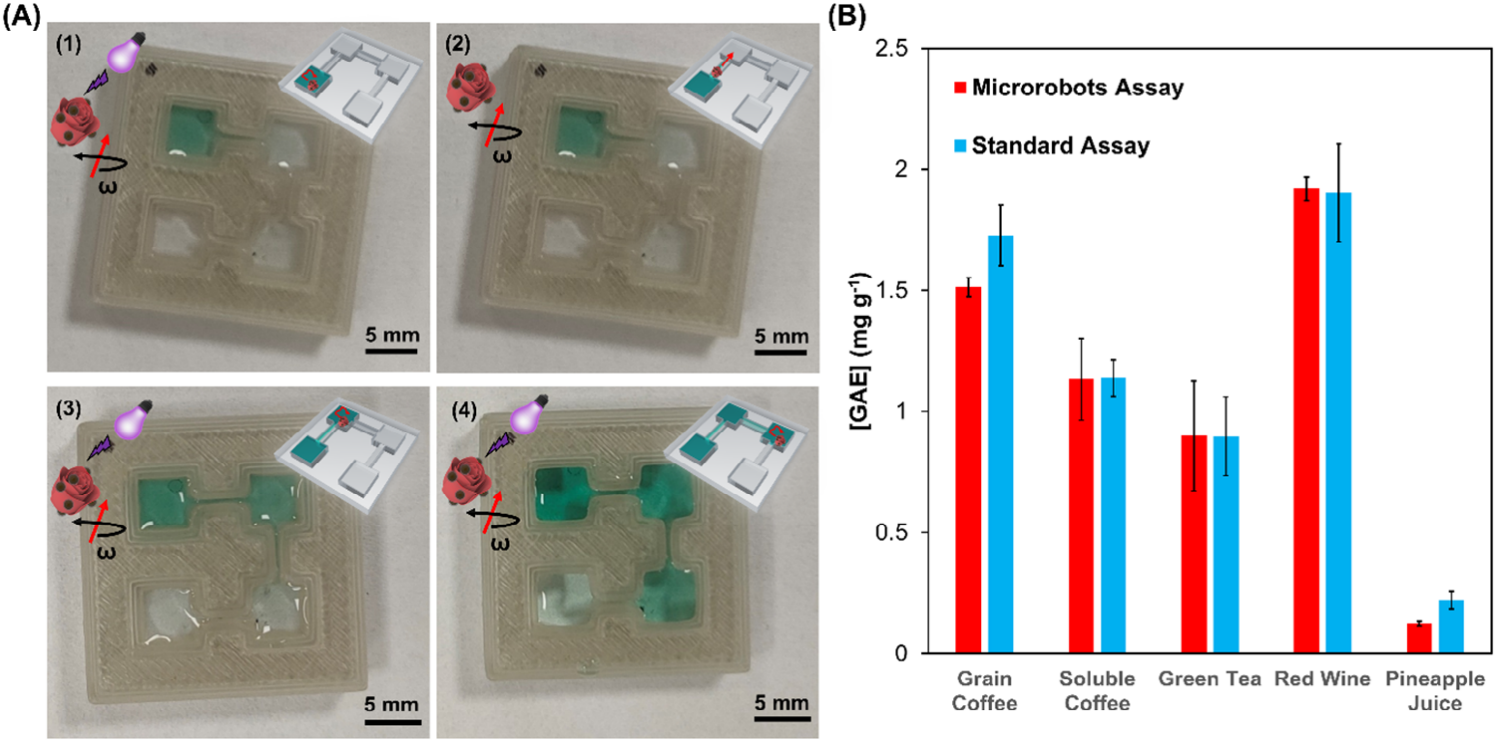
A) Images from the automated antioxidant activity analysis platform after microrobot actuation for different situations: (1) magnetic actuation following a random trajectory in the first reservoir after 20 min of light irradiation to perform the photocatalytic assay in the reservoir, (2) microrobots traveling between the first and second reservoirs under a unidirectional rotating magnetic field pointing to the second reservoir, (3) following the photocatalytic assay for antioxidant activity determination at the second reservoir, (4) following the photocatalytic assay for antioxidant activity determination at the third reservoir. B) Comparison of antioxidant activity determination between the automated microrobots assay described in this work and the standard fading ABTS^+·^ method for the different beverage samples.

The rotating magnetic field frequency and the times used for the automation program for antioxidant activity assay were optimized to obtain reproducible results in the four reservoirs. Figure S5A (Supporting Information) shows the effect of rotating magnetic field frequency on ABTS oxidation. ABTS oxidation improved from 2 Hz to 5 Hz frequency due to the enhanced intermixing with the microrobot speed (see Figure 3). However, 7 and 10 Hz rotating frequencies showed lower ABTS oxidation rates, probably associated to the microrobots agglomeration at higher frequencies. A 5 Hz frequency was chosen to perform the automated antioxidant activity assay because under these conditions ABTS was completely oxidized in a shorter time (20 min). Figure S5B (Supporting Information) shows the optimization of microrobots’ traveling time between reservoirs. As can be seen, constant ABTS^+·^ absorbance in all the reservoirs was obtained after only 20 min of traveling time. Shorter times produced a reduction of ABTS^+·^ signal after each reservoir due to the microrobots’ losses in each step. **Figure** **5**A shows images of the automated antioxidant activity assay in the absence of antioxidant compounds or samples. As can be seen, ABTS^+·^ green color evolution occurred in each reservoir after UV light irradiation independently. Only when Bi_2_O_2_CO_3_@Fe_3_O_4_ microrobots were in the corresponding reservoir did ABTS oxidation take place. Moreover, no significant ABTS^+·^ diffusion between reservoirs was observed during the automated antioxidant activity assay.

Finally, the antioxidant activity of several beverage samples was tested using the automated antioxidant activity assay developed in this work. Beverage samples contained different antioxidant compounds, hence, sample analysis results were referred to gallic acid equivalent (GAE) antioxidant activity. The low LOQ of the antioxidant activity assay based on Bi_2_O_2_CO_3_@Fe_3_O_4_ microrobots and the high content of antioxidant compounds in the samples allowed for direct colorimetric analysis without interference from the samples’ intrinsic color by straightforward dilution. The obtained results by the automated antioxidant activity assay were compared to a standard ABTS^+·^ fading method.^[^ ^43^ ^]^ Figure 5B shows the sample analysis results employing the automated microrobots platform and comparison to the standard method. A perfect correlation between the two methods for soluble coffee, green tea, and wine samples was obtained while grain coffee samples and pineapple juice showed slight differences, however, both methods showed the same order of magnitude of the samples’ antioxidant activity (**Table** **1**). The good results correlation between the two methods showed the potential of Bi_2_O_2_CO_3_@Fe_3_O_4_ microrobots to shorten and to automate quality analysis for food samples. The automated antioxidant activity assay presented in this study analyzes four samples in 140 min, significantly faster than the 240 min required by the standard method (excluding the overnight preparation of the ABTS+· standard solution).

**Table 1:** Sample analysis results employing the automated microrobots platform and comparison to a standard method (number of replicates per sample, *n* = 4).

Sample	Absorbance [a.u.]	[GAE] calibration [µM]	Dilution factor	[GAE] sample [mg g^−1^]	[GAE] sample [mg g^−1^]^a)^
Soluble coffee	0.18 ± 0.05	33 ± 5	1:200	1.1 ± 0.2	1.13 ± 0.08
Grain coffee	0.06 ± 0.01	44 ± 1	1:200	1.51 ± 0.03	1.7 ± 0.1
Green tea	0.25 ± 0.07	26 ± 6	1:200	0.9 ± 0.2	0.9 ± 0.2
Pineapple juice	0.49 ± 0.04	7.2 ± 0.5	1:100	0.12 ± 0.01	0.22 ± 0.03
Red wine	0.24 ± 0.01	28.2 ± 0.7	1:400	1.91 ± 0.05	1.9 ± 0.2

^a)^ Sample Analysis Results from the Standard Method^[^ ^43^ ^]^.

The antioxidant activity of our assay has been compared to similar procedures using static catalysts, such as nanoparticles, developed in recent years (**Table** **2**). As can be seen, all procedures have been used in beverage samples' antioxidant activity assessment with similar performance (LOQ and analysis time). However, automatization has been only achievable in this work owing to the incorporation of a magnetic material to the photocatalyst which allowed the magnetic actuation of the material.

**Table 2:** Antioxidant activity assays comparison using different nanozymes on beverages.

PHOTOCATALYST	SOURCE OF RADICALS	MAGNETIC ACTUATION	LOQ (µM) AS GAE	ASSAY TIME (MIN)	REFS.
CU‐CN	H_2_O	No	2.8	30	[45]
CU‐COO	H_2_O_2_	No	2.4	15	[46]
N‐MN_3_O_4_	H_2_O_2_	No	5	10	[47]
BI_2_O_2_CO_3_@FE_3_O_4_	H_2_O	Yes	2.8	20	This work

Moreover, colorimetric antioxidant activity assays are commonly used because they are easier to carry out, the costs are low, and the time required by the analysis is relatively short. However, real‐time analysis is not possible to achieve using such assays. To solve this drawback electrochemical sensors have been developed to assess antioxidant activity in real time. Even though it is true that electrochemical (bio‐)sensing allows real‐time analysis, it does not compensate for the analysis cost increment, and the requirement of electrochemical instrumentation and personnel trained in such electrochemical techniques rarely used in food quality control.^[^ ^44^ ^]^

## 3 Conclusion

To accelerate antioxidant activity quality analyses, magneto‐photocatalytic Bi_2_O_2_CO_3_@Fe_3_O_4_ microrobots were obtained by electrostatic interaction between the positive surface‐charged Bi_2_O_2_CO_3_ and negatively charged Fe_3_O_4_/SiO_2_ particles. These microrobots exhibited a surface “walk” magnetic actuation under a rotating transversal magnetic field. Moreover, they exhibited suitable photocatalytic activity under UV (395 nm) light irradiation for radicals’ generation such as OH^·^ and O_2_^−·^. These radicals were employed to develop an antioxidant activity assay based on ABTS oxidation by these radicals and radicals’ scavengers contained in the samples to hamper such reactions. Magnetic actuation of Bi_2_O_2_CO_3_@Fe_3_O_4_ microrobots reduced the time needed for the antioxidant activity assay due to enhanced intermixing of the solution. Moreover, magnetic actuation of the microrobots was employed to automate the assay on a 3D‐printed platform to minimize human interaction. Automated antioxidant activity sample analyses showed an excellent correlation to the standard ABTS^+·^ fading method and a significant decrease in analysis time (from 240 to 40 min per sample). All in all, we have demonstrated how magneto‐photocatalytic microrobots can be designed and employed for automated analytical assays to accelerate food quality/fraud analysis and meet the growing demand for such analysis, particularly by emerging economies.

## 4 Experimental Section

### Chemicals and Materials

Bismuth nitrate (abrc GmbH, Cat. AB119489), citric acid (Penta, Cat. 2 203 220 317), urea (Sigma‐Aldrich, Cat. U5128), iron(III) chloride hexahydrate (Sigma‐Aldrich, Cat. 236 489), trisodium citrate dihydrate (Sigma‐Aldrich, Cat. S4641), sodium acetate (Sigma‐Aldrich, Cat. S2889), ammonium hydroxide solution (28 wt%, Sigma‐Aldrich, Cat. 1 054 231 011), tetraethyl orthosilicate (TEOS, Sigma‐Aldrich, Cat. 131 903), ABTS (Thermo Scientific, Cat. J65535.06), gallic acid (Sigma‐Aldrich, Cat. 398 225), potassium persulfate (Sigma‐Aldrich, Cat. 216 224), “transparent” PLA filaments (clear 1.75 PLA, Prusa).

### Bi_2_O_2_CO_3_ Microparticles Synthesis

Bi_2_O_2_CO_3_ microparticles were synthesized following a previously reported hydrothermal synthesis method with small modifications.^[^ ^28^ ^]^ Briefly, 2.75 g of Bi(NO_3_)_3_ and 2.38 g of citric acid were mixed in 60 mL of ultrapure water. Then, the solution was sonicated for 1 h in an ultrasound bath and vigorously stirred for 3 h in a magnetic stirrer. Subsequently, 1.02 g of urea was added to the solution and stirred for 30 min at room temperature. After, the suspension was transferred into an 85 mL Teflon‐sealed autoclave and maintained at 180 °C for 12 h, then rested to cool at room temperature. Finally, the precipitate was collected, washed with ethanol and ultrapure water, and dried at 60 °C overnight in an oven.

### Fe_3_O_4_/SiO_2_ Nanoparticles Synthesis

Fe_3_O_4_ nanoparticles were synthesized by a solvothermal method following the previously reported procedure.^[^ ^29^, ^30^ ^]^ Briefly, FeCl_3_·6H_2_O (0.54 g) and trisodium citrate (0.2 g) were dissolved in 20 mL of ethylene glycol, after, 1.2 g of sodium acetate was added. The mixture was vigorously stirred for 30 min and subsequently transferred to a Teflon‐lined stainless‐steel autoclave with a volume of 50 mL. The reaction was then allowed to proceed at 200 °C for 10 h. The resulting black precipitates were washed with ethanol and ultrapure water and dried at 60 °C overnight in an oven. The Fe_3_O_4_/SiO_2_ was prepared using a typical sol‐gel process.^[^ ^31^ ^]^ Briefly, a 600 µL of the aqueous solution of Fe_3_O_4_ nanoparticles (25 mg mL^−1^) was mixed with 4 mL of ethanol and 200 µL of ammonia solution (28 wt%). The mixture was dispersed well under sonication for 30 min. After that, 25 µL of TEOS were quickly injected into the mixture and the reaction sonicated for 4 h. The resulting hydroxyl‐terminated Fe_3_O_4_/SiO_2_ nanoparticles were washed with ethanol and ultrapure water and dried at 60 °C overnight in an oven for further use.

### Bi_2_O_2_CO_3_@Fe_3_O_4_ Microrobot Assembly

900 µL of Bi_2_O_2_CO_3_ microparticles suspension (5 mg mL^−1^) were mixed with 100 µL of Fe_3_O_4_/SiO_2_ nanoparticles suspension (0.1 mg mL^−1^). The suspension was stirred overnight and collected by a magnet to separate the Bi_2_O_2_CO_3_ microparticles without Fe_3_O_4_ nanoparticles attached. The resulting Bi_2_O_2_CO_3_@Fe_3_O_4_ microrobots were dried until use. In the same way, Bi_2_O_2_CO_3_ microparticles were mixed with bare Fe_3_O_4_ nanoparticles, but Bi_2_O_2_CO_3_@Fe_3_O_4_ microrobots were not obtained from this mixture.

### Microrobot Characterization

SEM images were obtained by a ZEISS EVO 15 microscope equipped with an Oxford Instrument EDS detector for EDS mapping. An acceleration voltage of 10 kV was employed to obtain the images. TEM images were obtained using a Jeol JEM‐2200FS (JEOL, Japan) equipped with an energy‐dispersive X‐ray spectrometer (EDS). FTIR analysis was carried out by a Nicolet iS50 FTIR spectrometer (Thermo‐Nicolet, USA) with a diamond ATR attachment and a DTGS detector. XRD analysis was done by an X‐ray diffractometer (Bruker, AXS D8, Germany) using Co as the anode source. Data were transformed into a Cu reference as an anode source for representation. The Zeta potential of the samples was analyzed by a Zetasizer Pro (Malvern Panalytical, UK). UV–Vis characterization was carried out by a UV–Vis spectrometer (Shimadzu, UV‐2600, Japan). Bi_2_O_2_CO_3_@Fe_3_O_4_ microrobot Tafel plots were obtained by a drop‐casting modification of working electrodes with the microrobots. Then, sweep voltammetry was performed between 1 V and −1 V in water with/without light irradiation (λ = 395 nm) using a potentiostat (Autolab, Netherlands) and a three‐electrode quartz cell (Ag/AgCl reference electrode, Pt counter electrode, and the working electrode are previously described).

### Magnetic Actuation Experiments

The magnetic manipulation setup consisted of three pairs of coils disposed in a triaxial orientation on a home‐made 3D‐printed framework.^[^ ^39^ ^]^ Inside the coils, a transversal rotating magnetic field was generated and used for magnetic actuation of the microrobots. The device was controlled by a home‐made generator/regulator with programmable intensities, trajectories, and frequencies. An inverted Olympus IX73 optical microscope equipped with a monochrome CMOS camera (Basler acA‐1920‐155um) was used to record the microrobots’ motion and trajectories. Videos were cut and analyzed using NIS‐Elements software.

### Samples Preparation

Green tea bags with lemon from the local market were used to prepare tea samples. 200 mL of boiling tap water was employed to extract the tea from the bag (5 min). 2 g of coffee beans were used to prepare the coffee grain sample. The coffee beans were added into a Delongi espresso machine coupled with a grinder to obtain the powder. Subsequently, the machine used 100 mL of hot tap water to extract the coffee. Soluble coffee was prepared using 1 g of soluble coffee from a local market in 50 mL of tap water. Pineapple juice and red wine were used as purchased from the local market.

### ABTS colorimetric Assay Using Photocatalytic Microrobots

A total final volume of 100 µL was employed for the colorimetric assay. UV–Vis spectra were recorded using a 96‐well plate in a Multiskan SkyHigh microplate spectrophotometer (Thermo Fisher Scientific, USA). In each assay were mixed: 10 µL of microrobots suspension (2.5 mg mL^−1^), 20 µL of ABTS solution (50 mM), the corresponding volume of a gallic acid mother solution (100 µM) to obtain the correct dilution for calibration, and ultrapure water to reach 100 µL. Then, the 96‐well plate was placed inside the magnetic manipulation setup and irradiated from the top with UV light (395 nm) using a home‐made diode lamp (25 mW m^−2^) for 20 min.^[^ ^48^ ^]^ During the assay, the magnetic microrobots were under a transversal rotating magnetic field (5 mT, 5 Hz) and followed random trajectories.

Sample analyses were performed in a similar way. In this case, the gallic acid solution was replaced by the corresponding volume of sample to dilute them. The optimal dilutions to carry out the antioxidant activity determination of the samples were: 1:200 for tea, grain coffee, and soluble coffee samples, 1:100 for pineapple juice samples, and 1:400 for red wine samples. Moreover, the photocatalytic reaction was done in a 3D‐printed microfluidic platform and the resulting supernatant solution was transferred to a 96‐well plate for UV–Vis recording. All data were then expressed as gallic acid equivalents.

### ABTS^+·^ Fading Colorimetric Standard Assay

Before the antioxidant assay, ABTS^•+^ was prepared by mixing an ABTS solution (7 mM) with 2.45 mM potassium persulfate.^[^ ^43^ ^]^ This mixture was allowed to react overnight at room temperature in the dark. ABTS^•+^ solution was stored in the dark until use. For calibration, 200 µL ABTS^•+^ solution was mixed with the corresponding gallic acid volume (10–100 µM) and ultrapure water to reach a final volume of 300 µL in the wells of 96‐well plates. Finally, UV–Vis ABTS^•+^ determination was performed after 240 min and when absorbance reached a stable value (data not shown). For sample analysis, the gallic acid solution was replaced by a sample solution to achieve the sample dilution described above. All data were then expressed as gallic acid equivalents.

### 3D‐printed Platform Design and Print

Two different platforms were designed: one semi‐transparent to record the videos and the other for the antioxidant activity analysis. The semi‐transparent platform was designed in student Fusion 360 software (Autodesk, USA). The platform consisted of four square reservoirs of dimension 5 mm x 5 mm connected by channels of 0.4 mm. The design had a depth of 1.9 mm and a base of 0.1 mm. Then, the platform was printed in transparent PLA by a Prusa i3 MK3 3D printer (Prusa, Czech Republic) at 220 °C for extrusion and 60 °C on the bed with layers of 0.1 mm at a slow speed to avoid holes in the first thin layer. However, PLA of this thickness started to leak after 15–30 min contact with water solutions. Hence, another non‐transparent platform (with the same reservoirs and channel sizes) was designed with a base thickness of 1 mm to avoid leakage during antioxidant activity assays. This platform had 8 mm depth to accommodate the 100 µL volume in the reservoirs without overflowing.

## Conflict of Interest

The authors declare no conflict of interest.

## Author Contributions

The manuscript was written through contributions of all authors. All authors have given approval to the final version of the manuscript.

## Supporting information



Supporting Information

Supplemental Video 1

Supplemental Video 2

Supplemental Video 3

Supplemental Video 4

Supplemental Video 5

Supplemental Video 6

Supplemental Video 7

Supplemental Video 8

Supplemental Video 9

## Data Availability

The data that support the findings of this study are available from the corresponding author upon reasonable request.
