# Supplementary material for: High‐throughput Photoactive Magnetic Microrobots for Food Quality Control
Source: Small Methods. 2025 Mar 11;9(7):2401952. doi: 10.1002/smtd.202401952 (PMC12285628; doi:10.1002/smtd.202401952)
Supplement: Supplementary file 1 — Supporting Information [file SMTD-9-2401952-s001.docx]

Supporting Information

High-throughput photoactive magnetic microrobots for food quality control

Roberto Maria-Hormigos,^a^ Carmen C. Mayorga-Martinez,^b^ Jeonghyo Kim,^b^ Martin Pumera^a,b,c,d^*

^a^ Future Energy and Innovation Laboratory, Central European Institute of Technology, Brno University of Technology (CEITEC-BUT), Purkyňova 123, Brno, 61200, Czech Republic

^b^ Advanced Nanorobots and Multiscale Robotics Lab, Faculty of Electrical Engineering and Computer Science, VSB - Technical University of Ostrava, 17. listopadu 2172/15, 70800, Ostrava, Czech Republic

^c^ Department of Chemical and Biomolecular Engineering, Yonsei University, 50 Yonsei-ro, Seodaemun-gu, Seoul, 03722, South Korea.

^d^ Department of Medical Research, China Medical University Hospital, China Medical University, No. 91 Hsueh-Shih Road, Taichung, 40402, Taiwan.

* e-mail: [pumera.research@gmail.com](mailto:pumera.research@gmail.com)


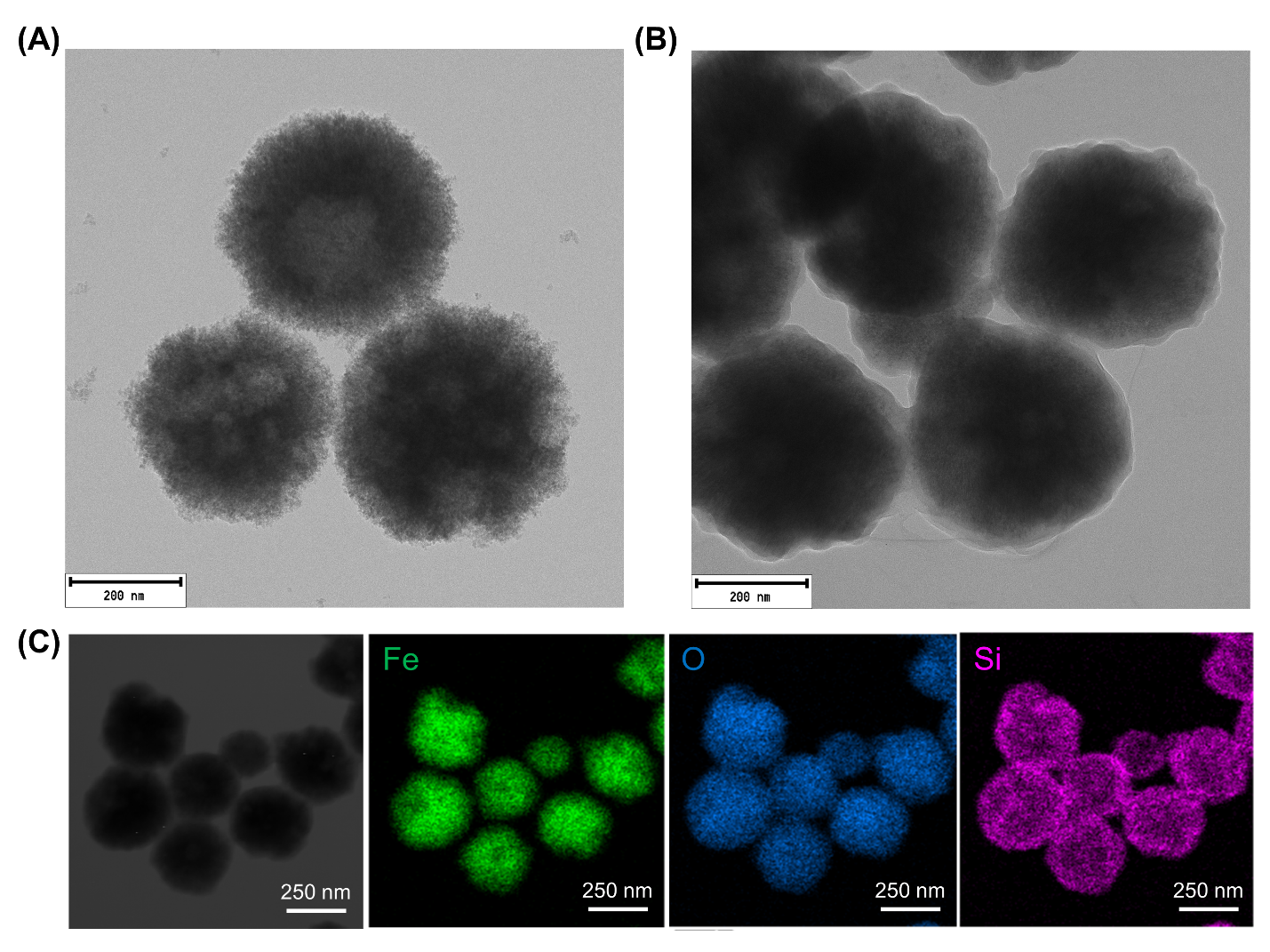


**Figure S1.** **(A)** TEM images of bare Fe_3_O_4_ nanoparticles. **(B)** TEM images of Fe_3_O_4_/SiO_2_ nanoparticles. **(C)** TEM/EDS elemental mapping of Fe_3_O_4_/SiO_2_ nanoparticles.


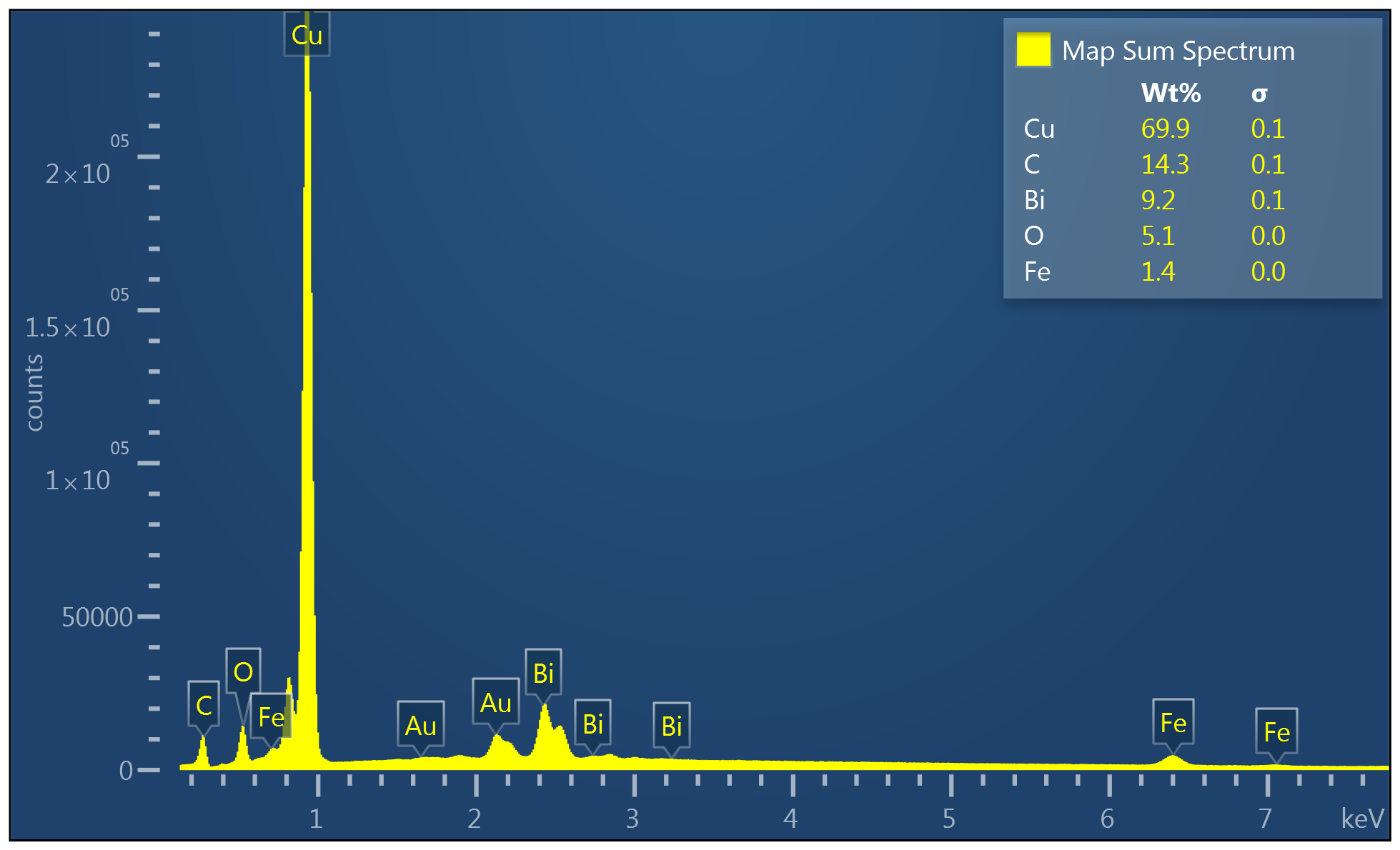


**Figure S2**. EDS spectrum of Bi_2_O_2_CO_3_@Fe_3_O_4_ microrobots.


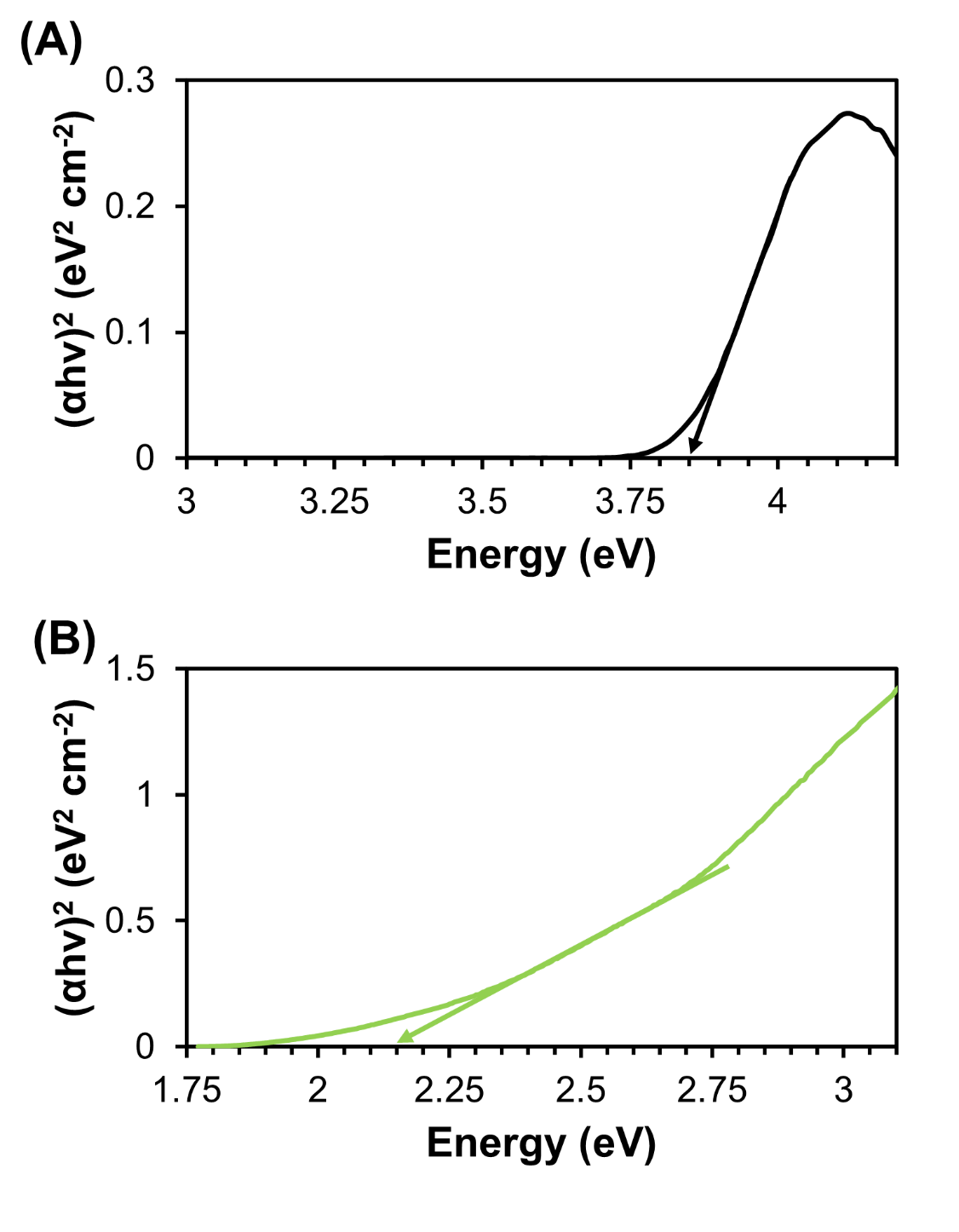


**Figure S3.** TAUC plots from UV-Vis spectra in Figure 2D of **(A)** bare Bi_2_O_2_CO_3_ microparticles and **(B)** Fe_3_O_4_/SiO_2_ nanoparticles. Arrows indicate the calculated band gap for the respective semiconductor materials.


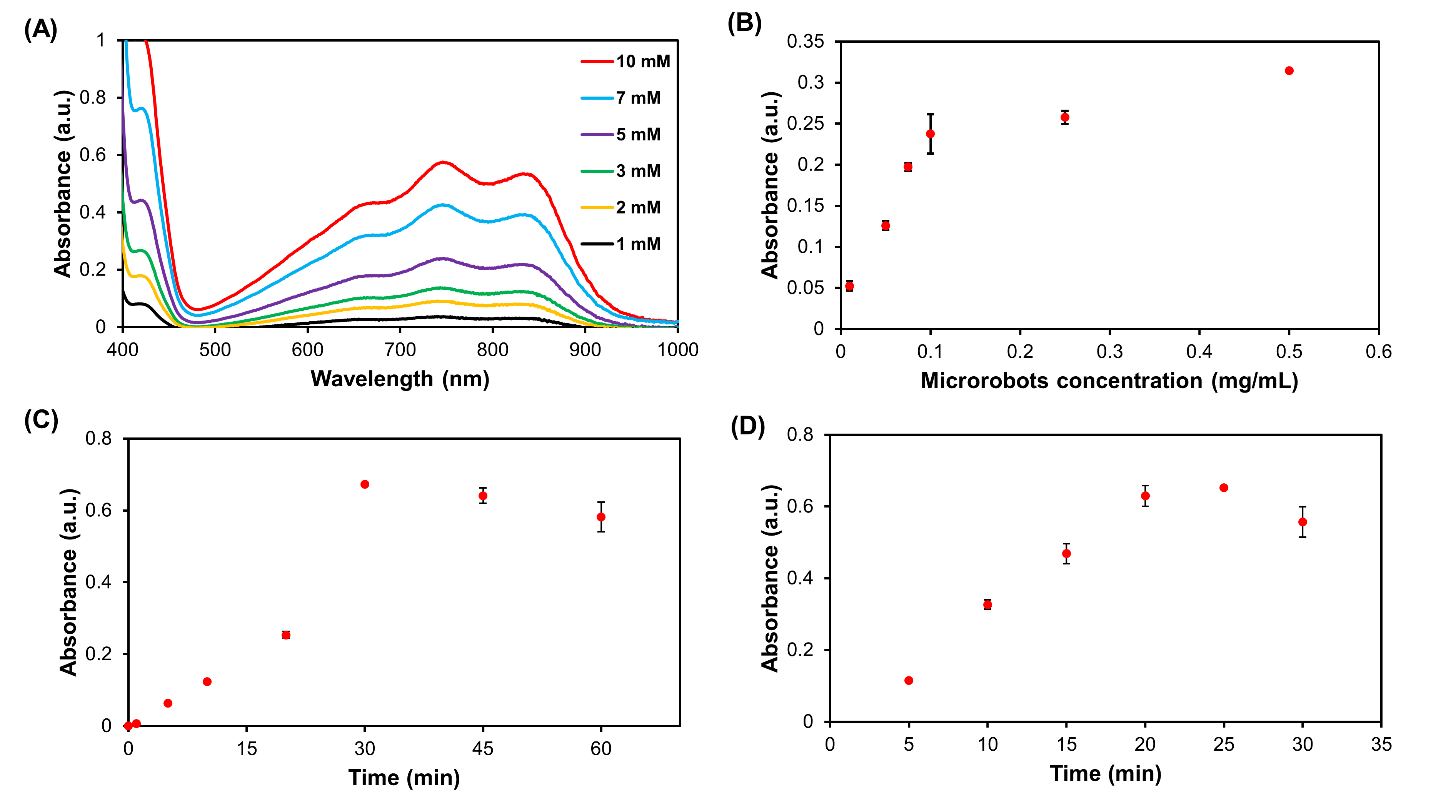


**Figure S4.** ABTS oxidation optimization by magnetic photocatalytic Bi_2_O_2_CO_3_@Fe_3_O_4_ microrobots. **(A)** ABTS concentration optimization; Experimental conditions: ABTS mixed overnight with 2.45 mM potassium persulfate. (*n* = 3). **(B)** Microrobots concentration effect; Experimental conditions: static microrobots after 20 min of light irradiation (λ = 395 nm), ABTS concentration 10 mM (*n* = 3). **(C)** Effect of irradiation time over static microrobots; Experimental conditions: 0.25 mg/mL microrobots irradiated 20 min (λ = 395 nm), ABTS concentration 10 mM (*n* = 3). **(D)** Effect of irradiation time over dynamic microrobots. Experimental conditions: Same conditions used in static experiments under a rotating magnetic field (5 mT, 5 Hz) (*n* = 3).


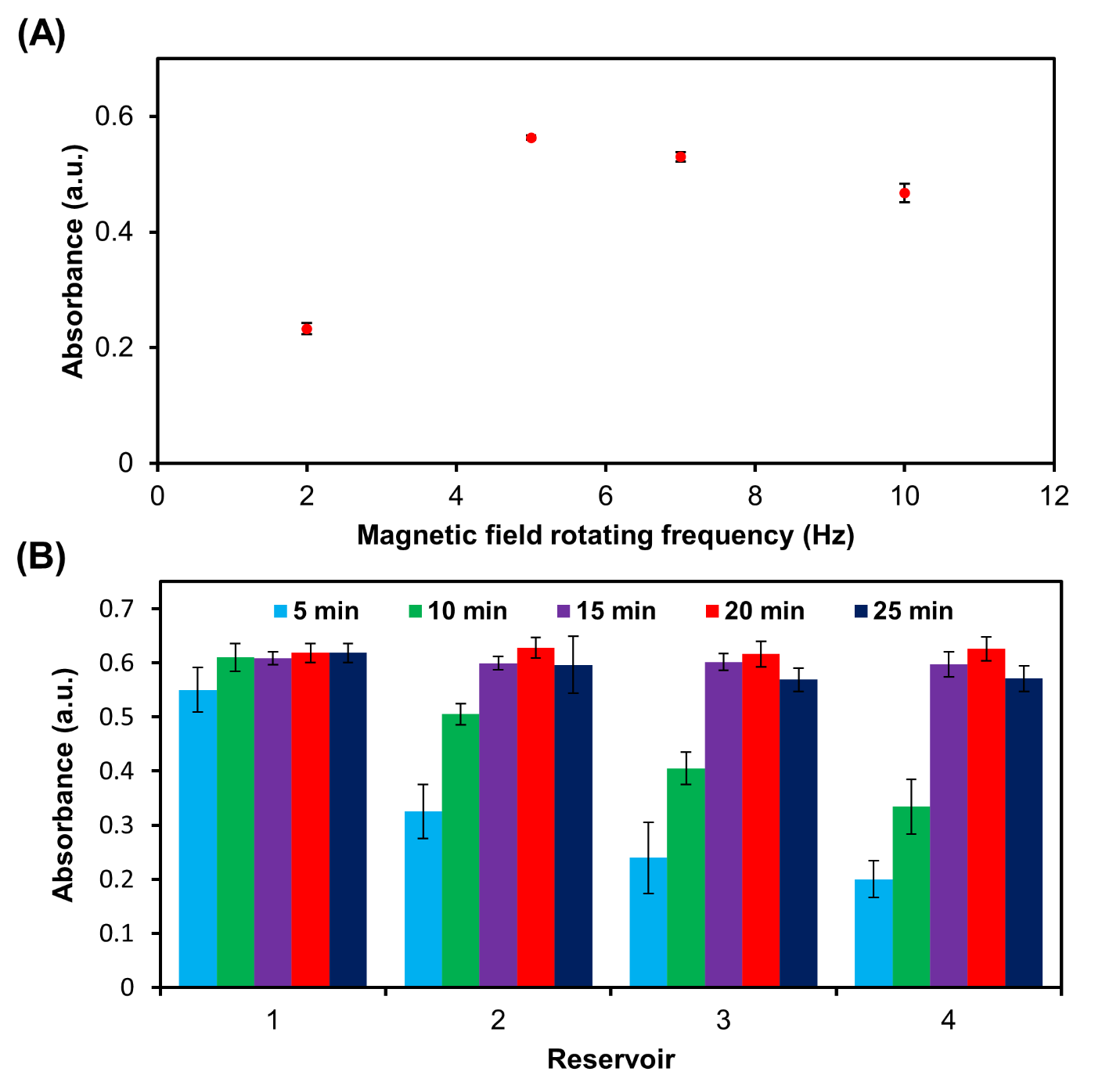


**Figure S5**. Automation optimization for antioxidant activity determination. **(A)** UV-Vis absorbance at 750 nm of 10 mM ABTS at different magnetic field rotating frequency; Experimental conditions: Bi_2_O_2_CO_3_@Fe_3_O_4_ microrobots concentration 0.1 mg/mL, light irradiation 20 min (λ = 395 nm), magnetic field intensity 5 mT (*n* = 4). **(B)** UV-Vis absorbance at 750 nm of 10 mM ABTS in each reservoir after different microrobots traveling time between reservoirs; Experimental conditions: microrobots 0.1 mg/mL, light irradiation 20 min (λ = 395 nm) in each reservoir, magnetic field 5 mT, 5 Hz (*n* = 3).
